# Fontan-Associated Hepatocellular Carcinoma: Case Report and Management Dilemmas

**DOI:** 10.7759/cureus.99609

**Published:** 2025-12-19

**Authors:** Bushra Mohandes, Salama Almheiri, Hajer Saleh Busharar, Essa M Aleassa, Ali Yammahi

**Affiliations:** 1 General Surgery, Mohamed bin Rashid University of Medicine and Health Sciences, Dubai Health, Dubai, ARE; 2 General Surgery, Rashid Hospital, Dubai Health, Dubai, ARE; 3 Hepatopancreatobiliary and Transplant Surgery, Digestive Disease Institute, Cleveland Clinic Abu Dhabi, Abu Dhabi, ARE; 4 Laparoscopic and Bariatric Surgery, Rashid Hospital, Dubai Health, Dubai, ARE

**Keywords:** congenital heart disease, fontan-associated liver disease, fontan procedure, hepatocellular carcinoma, non-cirrhotic hcc

## Abstract

Fontan-Associated Liver Disease (FALD) is a recognized complication post-Fontan procedure in surviving patients as a result of the long-term pathophysiological effects of elevated central venous pressure (CVP) on a hepatocellular level. While liver cirrhosis can occur, the risk of developing hepatocellular carcinoma (HCC) may develop without any histological or radiological evidence of cirrhosis. We describe the case of a 24-year-old male with a history of hypoplastic left heart syndrome, status post-Fontan procedure 16 years ago, and von Willebrand disease, who presented with mild right upper quadrant abdominal pain and fever. A CT scan was done on presentation and showed multifocal hepatic lesions with radiological features of HCC with no evidence of cirrhosis. Fontan patients are at higher risk of developing HCC even without cirrhosis. Increased awareness among physicians who are routinely involved with patients and initiating a structured surveillance program for this high-risk population is recommended.

## Introduction

The Fontan procedure is an established palliative treatment for patients with single-ventricle congenital heart disease, such as hypoplastic left heart syndrome. The procedure mainly includes bypassing the right ventricle by anastomosis of the right atrium to the pulmonary artery [1]. Recently, the percentage of adult survivors has gradually increased, reaching above 80% [1]. As a result, long-term complications of the Fontan circulation, including a distinct hepatic pathology termed Fontan-associated liver disease (FALD), are increasingly recognized [2]. The Fontan circuit relies on passive systemic venous return in the absence of a subpulmonary ventricle, resulting in chronically elevated central venous pressures and reduced cardiac output. This abnormal hemodynamic state leads to sinusoidal congestion, hepatocellular atrophy, and progressive fibrosis [3]. FALD encompasses this spectrum, ranging from mild congestion to cirrhosis and, rarely, hepatocellular carcinoma (HCC).

Recent studies suggest that the majority of adult Fontan patients develop some degree of hepatic fibrosis over time, even in the absence of traditional hepatic risk factors. HCC is a rare complication in this population, with reports stating a prevalence of approximately 4.5 to 5% [1,4], which has been attributed to the inevitable formation of hepatic fibrosis in this population.

The management of HCC in Fontan patients has unique challenges due to the altered hepatic architecture, cardiac dysfunction, and limited options for definitive therapies such as resection or transplantation. Other options include radiofrequency ablation (RFA) or transcatheter arterial chemoembolization (TACE), but data is limited to small case series. Here, we present the case of a 24-year-old male with a history of left hypoplastic heart disease status post-Fontan procedure and von Willebrand disease, who was found to have multifocal HCC without liver cirrhosis. This case emphasizes crucial factors to take into account when identifying, classifying, and managing HCC in patients with complicated congenital heart disease.

## Case presentation

A 24-year-old man who had a history of von Willebrand disease and hypoplastic left heart syndrome status post Fontan surgery presented at the emergency department complaining of fever, nausea, and continuous abdominal pain in the right upper quadrant. He denied experiencing jaundice, pruritus, urinary symptoms, vomiting, or changes in bowel habits. Upon examination, his vital signs were as follows: blood pressure of 129/74 mmHg, respiratory rate of 18 breaths per minute, heart rate of 104 bpm, and temperature of 38.5°C. Abdominal examination revealed a soft abdomen with mild tenderness in the right upper quadrant. The liver edge was palpable approximately 8 cm below the right costal margin. Labs upon admission showed deranged liver enzymes (as shown in Table 1).

**Table 1:** Labs upon admission AFP: alpha-fetoprotein; CRP: C-reactive protein; GGT: gamma-glutamyl transferase; ALT: alanine aminotransferase

Test	Result	Reference range—units
AFP	3,894	<5.8 IU/mL
CRP	23.3	
Liver Function Test		
Bilirubin	1.92	0-1.2 mg/dL
Alkaline phosphatase	203	40-129 U/L
ALT	81	0-41 U/L
Total protein	7.5	6.6-8.7 g/dL
Albumin	4.6	4.4-5.1 g/dL
GGT	138	8-61 U/L
CBC		
Hb	15.6	13-17 g/dL
WBC	6.2	3.6-11 10^3/uL
Platelets	293	150-410 10^3/uL
Urea and electrolytes		
Sodium	136	136-145 mmol/L
Potassium	4.7	3.4-4.5 mmol/L
Urea	34	12-40

He had presented four days earlier with similar but less severe symptoms, which he reported had been ongoing for two months. At that time, an abdominal ultrasound demonstrated a large, well-demarcated lesion in segment VIII of the right hepatic lobe. He was discharged with analgesia and a scheduled follow-up CT scan. However, the development of a fever prompted his earlier return to the hospital. His past medical history was notable for mild tricuspid and aortic regurgitation and mild ventricular systolic dysfunction. Surgical history included a Stage I Norwood procedure with Blalock-Taussig shunt at birth, Stage II Norwood at one year, and total cavopulmonary connection (TCPC) at age three via an 18 mm extracardiac fenestrated conduit. Fenestration was closed at age eight using a percutaneous, transcatheter occlusion device. He had also undergone a subinguinal varicocelectomy two years prior.

**Figure 1:**
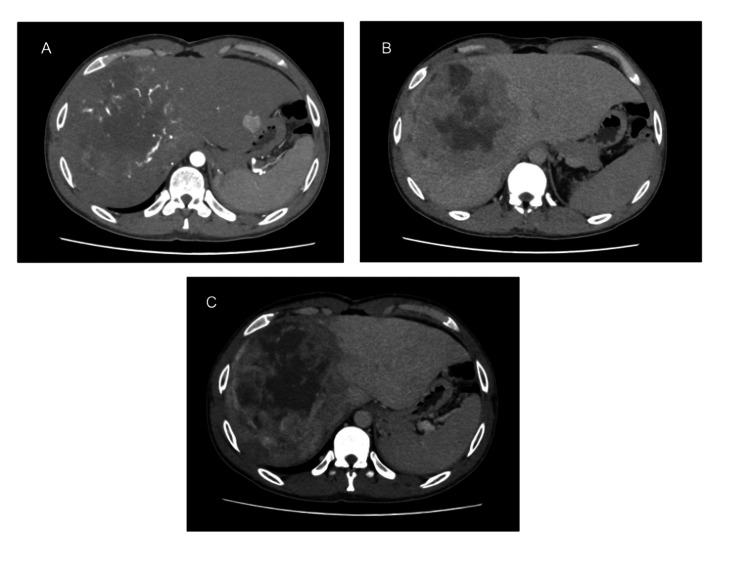
Triple phase axial CT images Triple phase axial CT images (A—arterial phase, B—delayed phase, C—venous phase) demonstrating a large hepatic mass in segment VIII with arterial phase enhancement, central necrosis, abnormally dilated feeding arteries, early venous washout, and delayed peripheral enhancement. A second lesion is seen in segment II of the left hepatic lobe, measuring 2.2 × 1.9 cm, with arterial enhancement and subtle delayed washout.

The patient was admitted and planned for further diagnostic evaluation. Laboratory investigations revealed an elevated alpha-fetoprotein (AFP) level of 3894 ng/mL. A large hepatic mass in segment VIII of the abdomen was seen on a contrast-enhanced CT scan. The mass had characteristics of hepatocellular carcinoma (HCC), including arterial phase enhancement, central necrosis, abnormally dilated feeding arteries, a fat component, early venous washout, and delayed peripheral enhancement (Liver Imaging Reporting and Data System (LI-RADS) 5). In segment II of the left hepatic lobe, a second lesion measuring 2.2 × 1.9 cm was identified (LI-RADS 4). It was suspicious for HCC due to the arterial enhancement and mild delayed washout (Figure 1).

**Figure 2:**
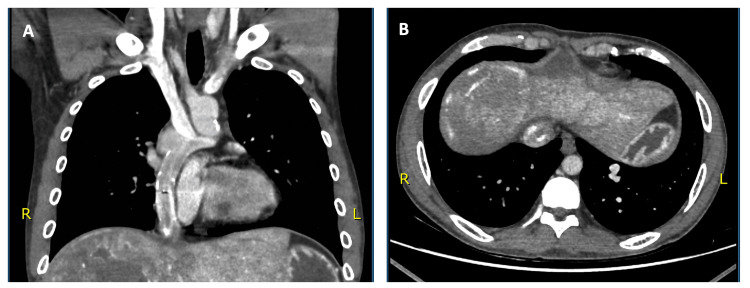
contrast-enhanced CT of the chest A: Coronal image showing anomalous systemic and pulmonary venous return. B: Axial image showing a vascular malformation measuring 3 × 1.4 cm noted at the base of the left lung.

A contrast-enhanced CT of the chest showed no evidence of pulmonary metastases but revealed findings consistent with previous congenital cardiac surgery, including anomalous systemic and pulmonary venous return. Additionally, a vascular malformation was noted at the base of the left lung, measuring 3 × 1.4 cm, composed of tortuous vessels draining into the pulmonary circulation (Figure 2).

The case was discussed in a multidisciplinary meeting involving hepatobiliary surgery, radiology, pathology, oncology, and congenital cardiology. It was agreed that the patient should be referred to medical oncology to evaluate the feasibility of initiating chemotherapy or immunotherapy. Concurrently, a referral was made to a hepatobiliary center for definitive management planning. Given his underlying Fontan physiology, he was referred to a congenital cardiologist to assess for possible surgical clearance to proceed with liver transplantation only or to opt for a combined liver-heart transplantation. The patient was subsequently discharged and transferred to the hepatobiliary center.

At the referral center, the patient was further investigated for treatment options. The lesion in segment 2 was biopsied and noted to be a focal nodular hyperplasia rather than an HCC, which gave the patient some hope for surgical resection, deeming it a single-lesion disease. Moreover, when the patient underwent a thorough congenital cardiac assessment, his heart was not deemed to be at a stage where he requires heart transplantation. Unfortunately, due to the lack of a comprehensive congenital heart disease service, it was unsafe to proceed with the right hepatectomy at the referral center in the United Arab Emirates (UAE). Therefore, the patient was referred to the appropriate center abroad for further definitive care.

## Discussion

The case highlights the correlation of a congenital single-ventricle physiology leading to FALD and, more rarely, HCC. Several studies have shown that hepatic fibrosis and cirrhosis are almost universal findings in patients with long-standing Fontan circulation, even in the absence of the typical hepatic risk factors [5]. However, unlike classical etiologies of HCC, Fontan-associated HCC may arise even in non-cirrhotic livers, such as in our case. This is likely due to a unique combination of low-flow hepatic perfusion, venous stasis, and hypoxia-induced oxidative stress [6]. These contradictory findings prompt the hypothesis that the Fontan circulation itself is a carcinogenic environment, particularly when fibrosis is already established.

In this case, the markedly elevated alpha-fetoprotein (AFP) level and imaging findings were characteristic of HCC, deferring the need for confirmation by histopathology [4]. Additionally, venous congestion and von Willebrand disease in this patient would increase the risk of procedural complications such as bleeding, especially in the setting of coagulopathy common in FALD.

The treatment options for HCC in Fontan patients are very limited. Surgical resection may be feasible in selected cases with preserved Fontan function; however, most patients have reduced physiologic reserve and portal hypertension, both of which increase operative risk. Liver transplantation is theoretically curative but presents challenges due to cardiac comorbidities in such patients [7]. Consideration for simultaneous heart transplantation should be in place if the liver transplant option is addressed. While simultaneous heart and liver transplantation has been performed for other pathologies in the past, it is imperative to appreciate the extra level of complexity that exists in patients with congenital heart disease, especially after already having had surgical correction in the past. The multidisciplinary team needs to be ready to deal with such complex scenarios perioperatively.

As for surgical resection of the liver lesion, a previous case report by Hur et al. reports HCC of 4 cm in size in a patient 30 years post-Fontan procedure, which was managed with segmentectomy without evidence of recurrence in a 3-year follow-up [8]. A special consideration in such cases is the lack of ability to run the central venous pressure low, as is the case in most liver resection procedures, due to the unique hemodynamic requirements to keep the systemic circulation well hydrated. This, along with the venous congestion that already exists in Fontan-associated liver disease patients, makes the likelihood of blood loss higher than usual. Such issues should be anticipated and well planned for.

In this case, the patient is being evaluated for a heart transplant prior to any liver-directed therapy, aiming to optimize his cardiopulmonary function and downstage the tumor burden with locoregional therapies. Although combined heart-liver transplantation is considered the ideal approach in most cases, certain considerations must be thoroughly assessed for that to happen. First of all, the identification of a center with congenital cardiac services, including multiorgan transplantation. Second, the eligibility of the patient for transplantation of both organs. Third, there is the issue of organ scarcity in the UAE, let alone identifying appropriate organs simultaneously [9]. Unfortunately, this case was considered ineligible for liver transplantation due to the large size of the HCC beyond the well-established criteria [10].

Surveillance strategies for this population are still evolving. Many centers now advise routine AFP monitoring and hepatic imaging beginning in adolescence, particularly when there is evidence of fibrosis or nodular transformation, regardless of the lack of consensus guidelines [1].

## Conclusions

This case highlights the importance of early imaging and detection, especially in patients with mild or ambiguous symptoms such as discomfort in the upper right quadrant or an unanticipated increase in transaminase levels. Management of HCC in this unique population remains challenging due to altered hemodynamics, limited hepatic reserve, and complex cardiac comorbidities that restrict surgical and transplant options.

Hepatologists, cardiologists, and primary care physicians need to be more aware of Fontan-associated HCC as the adult Fontan population grows. Careful perioperative planning, timely referral to interdisciplinary teams, and organized surveillance protocols are essential to improving outcomes in this high-risk population.
